# Assessment of renal volume by three-dimensional ultrasonography in pregnant bitches: an experimental study using virtual organ computer-aided analysis

**DOI:** 10.1186/1746-6148-8-102

**Published:** 2012-07-02

**Authors:** Débora Sartori Mendonça, Rafael Fontoura Fernandes Moron, André Luiz Louzada Maldonado, Edward Araujo, Luciano Marcondes Machado Nardozza, Antonio Fernandes Moron

**Affiliations:** 1 Department of Obstetrics, Federal University of São Paulo (UNIFESP), São Paulo, SP, Brazil

**Keywords:** Pregnant bitches, Renal volume, Two-dimensional ultrasound, Three-dimensional ultrasound, VOCAL

## Abstract

**Background:**

To assess and to compare the renal volume evolution in bitches during pregnancy by two-dimensional (2D) ultrasonography using the ellipsoid technique (volume = length x width x depth x 0.523) and three-dimensional (3D) ultrasonography using the Virtual Organ Computer-aided AnaLysis (VOCAL) method. A longitudinal prospective study was performed with 17 normal Golden Retrievers bitches during pregnancy from heat to the last third of gestation. The ultrasound scans were performed by two veterinarians. The left and right kidneys were assessed in three moments (day 0 = non-pregnant bitches; days 1^st^ to 20^th^ of pregnancy and days 21^st^ to 40^th^ of pregnancy) by three techniques (ellipsoid; VOCAL 12° and VOCAL 30°). For reproducibility calculations, we used the intraclass correlation coefficient (ICC).

**Results:**

The inferential result of the volumes in ANOVA revealed the interaction effect between side and moment (p = 0.009). The 3D techniques showed, in average, the same renal volumes (p = 0.137) regardless of the side and moment. Considering the right side, the renal volume in the day 0 was smaller than the day 21^st^ to 40^th^ (p = 0.029). Considering the left side, the renal volume at day 0 was smaller than the day 1^st^ to 20^th^ (p = 0.020) and day 21^st^ to 40^th^ (p = 0.007). It was found good intra observer reproducibility (ICC > 0.9) and none of the three techniques showed a good inter observer reproducibility (ICC < 0.7).

**Conclusion:**

The renal volume bitches by 3D ultrasonography using the VOCAL method (12° and 30°) had good correlation with the volume obtained by 2D ultrasonography method.

## Background

Currently, the prenatal ultrasound has become a constant routine in the veterinary diagnostic centers. The benefits allow us to plan an adequate management during the duration of pregnancy, to predict the most probable date of birth, to evaluate the fetal viability and the development and best date to perform an elective cesarean section. In contrast with the human medicine, that uses the fetal biometry, the veterinary medicine uses the morphological and embryonic annexes analysis in order to predict the gestational age and gestational monitoring. The length of canine pregnancy ranges between 62 and 64 days, but can occur in some occasions with a range between 58 and 71 days [1]. Despite all the ultrasonography advantages, there is little information available regarding standardization of dimensions and sonographic anatomy aspects of several organs in adults and fetuses in a variety of animal species of different breeds.

The three-dimensional (3D) ultrasonography, available in medicine for over ten years enabled great advances in the area of imaging diagnostic, mainly in obstetrics and gynecology. The 3D ultrasonography facilitates the volumetric study of organs and structures, besides it provide a third image plane, the coronal plane, which allows more precisely volumetric calculation mainly those of irregular shapes [2]. The 3D ultrasonography is still a new technique in veterinary medicine and few are the scientific papers that report its experimental use.

During the pregnancy course occurs maternal hemodynamic modifications including the changes in maternal renal volumetry. The kidneys grow about 1 – 1.5 cm for most renal vasculature. The renal volume increases 30% due an increase in renal plasmatic flow and glomerular filtration rates result**i**ng an increase in cardiac debt, decrease in renal vascular resistance and increase of the seric hormonal levels. The canine renal ultrasonography provides valuable anatomical information regarding the size, shape and internal architecture of the kidneys, which can be obtained even in the presence of a poor renal function or abdominal fluid [3].

The assessment of renal volume by 3D ultrasonography is the great importance in an attempt to bring a new comprehension about the maternal hemodynamic modifications during the course of the pregnancy and the application of this new method in dogs, besides comparing this new technology with the conventional two-dimensional (2D) ultrasonography.

## Results

Of the 19 bitches initially selected, 2 were excluded due the impossibility of performing the analysis because of advanced gestational age (over 40 days) and elevated number of fetuses (12 or more). These bitches showed puffy breathing pattern and excessive restlessness during the ultrasound scan and the fetal images overlapped in renal topography, which resulted in an unsatisfactory quality. For these reasons, the study was limited to the 40^th^ day of pregnancy.

The sample selected in this study was composed by 17 bitches with average of 2.9 ± 0.7 (SD – standard deviation) years, ranging from 2 to 4 years. It was found that 8 (47.1%) of those bitches weren’t pregnant. Among the 9 bitches remaining (52.9%), the number of fetuses ranged from 1 to 10. In the total number of bitches, 6 (35.3%) were in their first pregnancy. In the group of the 11 remaining bitches (64.7%), the number of previous pregnancies ranged from 1 to 4.

It were collected for this study 150 volumes by the first examiner (DSM) being 75 of the left kidney. The renal volume was assessed by 2D ultrasonography method (ellipsoid) in all of the cases, corresponding to a sensitivity of 100%. For the 3D ultrasonography method using the Virtual Organ computer-aided AnaLysis (VOCAL) 12 and 30, 73 volumes could be evaluated; corresponding to a sensitivity of 97.3%. 60 volumes of the right kidney could be assessed by 2D method, corresponding to a sensitivity of 80%. For the VOCAL 12 and 30 methods, 58 volumes were assessed, corresponding to a sensitivity of 96.6%.

The second examiner (RFFM) collected 124 renal volumes, being 62 of the left kidney. The renal volume was assessed by 2D method (ellipsoid) in all of the cases, corresponding to a sensitivity of 100%. For the VOCAL 12 and 30 methods, 57 renal volumes could be assessed, corresponding to a sensitivity of 91.9%. 48 volumes of the right kidney could be assessed by 2D method, corresponding to a sensitivity of 77.4%. For the VOCAL 12 and 30 methods, 44 volumes were assessed, corresponding to a sensitivity of 91.6%.

Table 1 shows the comparison of renal volume between both sides of the kidneys; ultrasonography technique (ellipsoid; VOCAL 30° and 12°) and moment of time (day 0; 1^st^ to 20^th^ days and 21^st^ to 40^th^ day). For this purpose, we selected the first measurement of the first examiner (DSM). The inferential result of ANOVA in renal volumes revealed the existence of an interaction effect between side and moment (p = 0.009), which means that the renal volume behavior during the three time moments for the right side, isn’t statically equal for the renal volume behavior during the three time moments for the left side. So, the comparison of both sides of the renal volume will depend on the moment, as well as the comparison of the renal volume in the moment will depend on the side. It wasn’t detected an interaction effect between side and technique (p = 0.193) and also between moment and technique (p = 0.917). The three techniques present, in average, the same renal volumes (p = 0.137) independently of the side and moment.

**Table 1:** Summary of measurements realized by the first examiner (DSM), according to the side, technique and moment

**Side**	**Technique**	**Moment**	**N**	**Average**	**Median**	**Minimum**	**Maximum**	**Standard deviation**
Right	2D	0 day	7	57.50	53.25	45.05	72.92	10.28
		1^st^ - 20^th^ days	7	56.05	54.40	42.82	78.30	10.73
		21^th^ - 40^th^ days	6	59.32	54.36	46.15	94.06	17.78
		Total	20	57.54	54.34	42.82	94.06	12.44
	VOCAL 30	0 day	7	58.70	58.46	41.37	74.08	10.37
		1^st^ - 20^th^ days	7	54.45	53.41	44.26	63.89	6.89
		21^th^ - 40^th^ days	6	57.89	57.77	44.79	73.72	9.77
		Total	20	56.97	55.52	41.37	74.08	8.82
	VOCAL 12	0 day	7	55.80	54.83	37.35	71.72	10.76
		1^st^ - 20^th^ days	7	50.95	53.16	39.10	62.21	7.68
		21^th^ - 40^th^ days	6	56.37	54.39	45.26	74.17	10.25
		Total	20	54.27	53.76	37.35	74.17	9.44
	Total	0 day	21	57.34	55.45	37.35	74.08	10.01
		1^st^ - 20^th^ days	21	53.82	53.53	39.10	78.30	8.44
		21^th^ - 40^th^ days	18	57.86	56.77	44.79	94.06	12.39
		Total	60	56.26	54.36	37.35	94.06	10.28
Left	2D	0 day	9	49.95	52.79	35.53	63.14	10.51
		1^st^ - 20^th^ days	7	54.22	52.02	42.83	71.43	10.13
		21^th^ - 40^th^ days	9	53.91	55.74	34.01	69.53	10.44
		Total	25	52.57	53.68	34.01	71.43	10.14
	VOCAL 30	0 day	9	54.53	53.11	41.41	75.51	10.66
		1^st^ - 20^th^ days	7	57.68	56.03	49.01	72.74	7.76
		21^th^ - 40^th^ days	9	55.67	57.31	42.21	64.29	7.78
		Total	25	55.82	56.03	41.41	75.51	8.65
	VOCAL 12	0 day	9	52.26	51.36	39.34	69.34	9.82
		1^st^ - 20^th^ days	7	54.8	54.28	47.17	65.87	6.06
		21^th^ - 40^th^ days	9	54.36	54.83	36.95	65.57	8.77
		Total	25	53.72	54.28	36.95	69.34	8.26
	Total	0 day	27	52.24	52.79	35.53	75.51	10.11
		1^st^ - 20^th^ days	21	55.56	55.55	42.83	72.74	7.89
		21^th^ - 40^th^ days	27	54.65	55.74	34.01	69.53	8.74
		Total	75	54.04	54.28	34.01	75.51	9.03
Total	2D	0 day	16	53.25	53.02	35.53	72.92	10.78
		1^st^ - 20^th^ days	14	55.13	54.34	42.82	78.30	10.07
		21^th^ - 40^th^ days	15	56.08	55.74	34.01	94.06	13.52
		Total	45	54.78	54.28	34.01	94.06	11.36
	VOCAL 30	0 day	16	56.36	56.39	41.37	75.51	10.40
		1^st^ - 20^th^ days	14	56.07	54.54	44.26	72.74	7.24
		21^th^ - 40^th^ days	15	56.56	57.31	42.21	73.72	8.36
		Total	45	56.33	55.55	41.37	75.51	8.64
	VOCAL 12	0 day	16	53.81	53.33	37.35	71.72	10.05
		1^st^ - 20^th^ days	14	52.88	53.72	39.10	65.87	6.94
		21^th^ - 40^th^ days	15	55.16	54.83	36.95	74.17	9.08
		Total	45	53.97	54.28	36.95	74.17	8.71
	Total	0 day	48	54.47	53.87	35.53	75.51	10.28
		1^st^ - 20^th^ days	42	54.69	54.28	39.10	78.30	8.12
		21^th^ - 40^th^ days	45	55.93	55.74	34.01	94.06	10.34
		Total	135	55.03	54.31	34.01	94.06	9.63

VOCAL: Virtual Organ Computer-aided AnaLysis; N: number of bitches in each gestational period.

Taking the right side in consideration, the renal volume in the day 0 was equal to which was found in the 1^st^ to 20^th^ gestational days (p = 0.987) and lower than at the 21^st^ to 40^th^ gestational days (p = 0.029). The renal volume found in the 1^st^ to 20^th^ gestational days was lower than at the 21^st^ to 40^th^ gestational days (p < 0.001). Taking the left side in consideration, the renal volume in the day 0 was lower than at the 1^st^ to 20^th^ gestational days (p = 0.020) and 21^st^ to 40^th^ gestational days (p = 0.007). The renal volume in the moment 1^st^ to 20^th^ gestational days was equal to the moment 21^st^ to 40^th^ gestational days (p = 0.508) (Table 2).

**Table 2:** **Comparison of the moments at 0 day, 1**^**st**^**- 20**^**th**^**days and 21**^**st**^**- 40**^**th**^**days of pregnancy for the right and left sides**

**Side**	**Conclusion**	**p**
Right	0 day = 1^st^ - 20^th^ days	0.987
	0 day < 21^th^ - 40^th^ days	0.029
	1^st^ - 20^th^ day s < 21^st^ - 40^th^ days	<0.001
Left	0 day < 1^st^ - 20^th^ days	0.020
	0 day < 21^st^ - 40^th^ days	0.007
	1^st^ - 20^th^ = 21^st^ - 40^th^ days	0.508

In relation to the method reproducibility, we observed good intraobserver reproducibility with ICC values closest to 1 (Table 3). For the interobserver reproducibility, a weak concordance was observed between the examiners with ICC values closest to 0. The examiners presented an reasonable concordance in the measure at: 1) right side, VOCAL 30°, day 0; 2) left side, ellipsoid technique, day 0; 3) left side, VOCAL 30°, 21^st^ to 40^th^ gestational days; and 4) left side, VOCAL 12°, day 0 (Table 4).

**Table 3:** **Estimates of intraclass correlation coefficients between 1**^**st**^**and 2**^**nd**^**measurements realized by the first examiner (DSM)**

	**ICC**	**Interval for the ICC of 95%**		**p**
right side, 2D, day 0	0.957	0.799	0.992	<0.001
right side, 2D, 1^st^ - 20^th^ days	0.973	0.850	0.996	<0.001
right side, 2D, 21^st^ - 40^th^ days	0.958	0.771	0.994	<0.001
right side, VOCAL 30, day 0	0.962	0.820	0.993	<0.001
right side, VOCAL 30, 1^st^ - 20^th^ days	0.889	0.481	0.983	0.002
right side, VOCAL 30, 21^st^ - 40^th^ days	0.968	0.824	0.995	<0.001
right side, VOCAL 12, day 0	0.943	0.739	0.990	<0.001
right side, VOCAL 12, 1^st^ - 20^th^ days	0.639	0.000	0.926	0.034
right side, VOCAL 12, 21^st^ - 40^th^ days	0.944	0.705	0.992	<0.001
left side, 2D, day 0	0.953	0.821	0.989	<0.001
left side, 2D, 1^st^ - 20^th^ days	0.889	0.539	0.980	0.001
left side, 2D, 21^st^ - 40^th^ days	0.786	0.340	0.946	0.002
left side, VOCAL 30, day 0	0.941	0.777	0.986	<0.001
left side, VOCAL 30, 1^st^ - 20^th^ days	0.879	0.505	0.978	0.001
left side, VOCAL 30, 21^st^ - 40^th^ days	0.734	0.227	0.932	0.006
left side, VOCAL 12, day 0	0.971	0.887	0.993	<0.001
left side, VOCAL 12, 1^st^ - 20^th^ days	0.954	0.785	0.992	<0.001
left side, VOCAL 12, 21^st^ - 40^th^ days	0.954	0.823	0.989	<0.001

VOCAL: Virtual Organ Computer-aided AnaLysis; 2D: two-dimensional ultrasonography; ICC: intraclass correlation coefficient.

**Table 4:** Estimates of intraclass correlation coefficients between the measurements realized by the first (DSM) and the second examiner (RFFM)

	**ICC**	**Interval for the ICC of 95%**		**p**
right side, 2D, day 0	0.501	0.331	0.909	0.106
right side, 2D, 1^st^ - 20^th^ days	0.484	0.000	0.928	0.138
right side, 2D, 21^th^- 40^th^ days	0.000	0.000	0.816	0.621
right side, VOCAL 30, day 0	0.765	0.009	0.972	0.024
right side, VOCAL 30, 1^st^ - 20^th^ days	0.000	0.000	0.806	0.639
right side, VOCAL 30, 21^th^ - 40^th^ days	0.180	0.000	0.912	0.356
right side, VOCAL 12, day 0	0.581	0.000	0.945	0.089
right side, VOCAL 12, 1^st^ - 20^th^ days	0.000	0.000	0.757	0.588
right side, VOCAL 12, 1^st^ - 20^th^ days	0.000	0.000	0.801	0.649
left side, 2D, day 0	0.631	0.000	0.912	0.027
left side, 2D, 1^st^ - 20^th^ days	0.334	0.000	0.899	0.233
left side, 2D, 21^th^ - 40^th^ days	0.454	0.000	0.858	0.097
left side, VOCAL 30, day 0	0.301	0.000	0.802	0.202
left side, VOCAL 30, 1^st^ - 20^th^ days	0.123	0.000	0.846	0.388
left side, VOCAL 30, 21^th^ - 40^th^ days	0.672	0.000	0.933	0.025
left side, VOCAL 12, day 0	0.736	0.185	0.940	0.008
left side, VOCAL 12, 1^st^ - 20^th^ days	0.000	0.000	0.727	0.533
left side, VOCAL 12, 21^th^ - 40^th^ days	0.000	0.000	0.570	0.698

VOCAL: Virtual Organ Computer-aided AnaLysis; 2D: two-dimensional ultrasonography; ICC: intraclass correlation coefficient.

## Discussion

The possibility of using the 3D ultrasonography as a imaging method in veterinary medicine would be extremely useful, especially because of its image quality and its volumetric results being in many matters compared to the magnetic resonance imaging (MRI) [4,5], because for the animals, the MRI and the computed tomography (CT) are necessary general anesthesia in order to be performed, which often prevents the patient be evaluated and/or diagnosed. The importance of volumetric measurement is related to follow the evolution of specific diseases and following of each individual patient, which is often done subjectively, through correlation with other anatomic regions [6], because of the difficulty in establishing reliable standard parameters in variety of species and breeds and by the difference between the conformation of the thorax and abdomen in animals of same weight. So, we considered of great importance new ways that allow this evaluation. These new image techniques should be easy and fast and with a good accuracy when performed.

In this study were performed three measurements by each examiner of every method using the average for volumetric calculation in resemblance to other described in the literature [7,8]. We used the ellipsoid formula for the 2D method. The length was assessed in the longitudinal plan and the width and depth in the axial plan [7,9]. Regarding the techniques, it was chose to be compared, the 2D and 3D ultrasonography methods using the VOCAL 12° and 30°. Several studies in human medicine also compared the rotation angles of VOCAL method, some of which indicate that the use of 30° is fast and has good efficacy [10] and others that indicate that a greater number of the rotation steps increase the accuracy of method [11,12].

In 5 bitches the right kidney volume couldn’t be calculated (2 non-pregnant – day 0; and 3 in 21^st^ to 40^th^ gestational period) resulting from the presence of gas in right kidney topography, probably by a failure in the requested fasting or aerophagia which corresponded to 15 of non-collected volumes (80% of success in the right kidney in relation to the left kidney) concerning the first examiner (DSM) which is consistent with other authors, due the relation between the right kidney with the descendent duodenum portion and ascending colon which cause bigger reflection and attenuation of the sound beam prejudicing the image formation [3,13]. All the remaining volumes (60) were submitted to the 2D method by the first examiner, having lost only of two volumes of the measurements relating to rejoinders by VOCAL 30° and 12° methods, with a success of 96.6% of the measurements related to the 100% of 2D technique.

In our study, the mean of renal volume (day 0) was respectively for the right and left of 57.34 cm^3^ and 52.24 cm^3^. This result indicates that the right kidney has a volume 8.9% bigger than the left kidney. This is a result that can be confronted and extrapolated with the results of other authors; cause indicates the renal volume of normal non-pregnant bitches. Some studies founded a significant difference in which the left kidney has a bigger volume than the right kidney and other ones did not find this difference [6,14]. The total renal volume was calculated as 3.5 mL/kg with a variation of 1.8 to 6.9 mL/kg [6] and taking in consideration that a Golden Retriever bitch weights an average of 30 kg, the value would be of 105 mL (54 – 207 mL) with very wide confidence level. In other study with dogs of 20.1 to 45 kg, the authors found an average of renal volume of 53.81 cm^3^[14], this result was similar to the present study. Thus, some authors claim that despite the correlation of the linear measurements by ultrasonography, the high standard deviation is a limitation [3]. Taking these limitations in consideration its use can be considered sufficiently precise for the clinic use in dogs. In this study, the standard deviation was lower in the observations by VOCAL, independently of the observer, side or moment. We believe that the differences between right and left renal volumes observed in this study occurred because of the short time among the assessments. The period of 20 days probably was enough to significant changing in the renal volume of pregnant bitches. Only future studies with a greater range among the ultrasound scans and greater number could reach definitive conclusions.

The difference was, in average, of 6.5% increase in renal volume during the pregnancy course, less significant than the 30% found in human medicine literature [15], although is a result unknown in bitches which should be investigated in a study with larger sample. These data indicate that there is a real necessity of studies to give a better understanding of the gestational physiology in animals, because we found renal volumetric alterations consistent with probable morphological and functional alterations during the gestation similarly to what happens with women, this subject should be further investigated aiming the bitch life quality besides in the commercial and economic viability of the pups.

The 3D ultrasonography techniques presented, in average, the same renal volumes independent of the side and moment. This result must be investigated in other study, because it was expected that the VOCAL 12° method had more accuracy than the 30°. This study did not find correlation between of renal volumes in the day 0 neither with the maternal age nor with the number of previous gestations. It was expected that sequential pregnancies could permanently increase the volume independently of side or technique.

There was little correlation between the first assessment of the first examiner (DSM) and the assessment of the second examiner (RFFM) with intraclass correlation coefficients near 0. It’s important to emphasize that these results, probably had occur because of the wide confidence intervals resulting from the low sample size. It should be noted that each examiner evaluated your own volume (reproducibility) differently of the many studies in human medicine in which the second examiner evaluates the collected volume by the first examiner (repeatability). Nowadays, the correct form to assess the reproducibility between different examiners is that the ultrasound scans are realized in different moments, with different conditions. When the ultrasound scans are realized in the same conditions, for example in the same volumes, the correct name is repeatability. The fact of our ultrasound scans were realized in different conditions becomes our results of high value to be used in the clinical practice. There is not until this moment any study regarding interobserver reproducibility of volumetric measurements by 2D and 3D ultrasonography in dogs.

3D ultrasonography presents some gains to be used in the practical clinic as the superiority of image; more accuracy t in volume calculate; short time to realized the exam and less operator-dependent than 2D ultrasonography. Our experience showed that the unsuccessful in the 3D volume scanning occurred in the same cases that the quality of the 2D wasn’t the ideal as has occurred with the lost right kidney volumes. The volumes had very good quality, however they weren’t equals to the MRI or CT image scans, because the animals were not anesthetized. Therefore, we observed that the volumetry obtained by 3D ultrasonography was similar to 2D ultrasonography and therefore with sufficient accuracy for the clinical practice, without the necessity of general anesthesia. Considering these observations, the volume calculation by VOCAL method proved to be useful and precise regarding to the conventional technique. We indicate the VOCAL 30 method because it presents best correlation among validity, reliability and realization time of the ultrasound scan.

As limitations of the 3D ultrasonography we can mention the necessity of training for the achievement of the volume and standardization of its collecting methods to prevent the loss of the spatial orientation of the organ or structure; decreased volume quality by the presence of movement which requires a cooperative patient or sedation in cases of restless animals; increased formatting and interpretation time of the stored volumes; optimized software configuration for the human body and relatively high equipment cost.

We believe that the assessment of renal volume of pregnant bitches by 3D ultrasonography using VOCAL can be used to monitor the renal function during the pregnancy, mainly in cases of chronic diseases as diabetes and arterial hypertension. These conditions usually can modify the normal renal function and the renal volume by 3D ultrasonography can detect early alterations.

## Conclusions

In summary, this was a preliminary and pioneer study in the assessment of renal volume in pregnant dogs. These initial results open new perspectives of studies that can compare the organs volumetry in sedated animals by the CT scan with the results obtained by 2D and 3D ultrasonography.

## Methods

Was performed a longitudinal prospective study, with 19 normal Golden Retrievers pregnant bitches in the period of April to August of 2010. This study was approved by the Research Ethics Committee of the Federal University of São Paulo (UNIFESP) under the number of 1859/09. The inclusion criteria were bitches with ages between 1 to 6 years old and docile behavior. The exclusion criteria were systemic pathologies like diabetes, arterial hypertension, fever; polycystic renal disease or another renal affection visualized during the realization of ultrasonography scans, beyond of renal volumes with low quality of its edges.

The ultrasonography scans were performed in the Golden Trip Kennel in the city of Embú das Artes, São Paulo, SP, Brazil. In this kennel, the dogs stay in big pickets separated in three groups by sex and age: puppies up to 6 months, puppies of 6 months to 1 year and adulthood. The pregnant bitches remain in individual bays. The maternity is located in an isolated house away of these places, were the matrix remains with the puppies until 45 days. With 45 days occurs the immunization and the puppies are directed to the pickets of the puppies up to 6 months. After the heat detection in the bitch, natural mating is performed three times on alternate days. The day of the first mating is considered the first gestational day. The animals were fed with ration twice a day and water *ad libitum*.

The ultrasound scans were performed by two veterinarians (DSM and RFFM), utilizing an equipment SonoAce 8000Live (Samsung Medison, Seoul, Korea) equipped with a volumetric convex transducer (2 – 5 MHz). Both examiners presented one year of experience in collecting volumes in dogs by 3D ultrasonography. For the calculations of the renal volume, we used the 2D ultrasonography by the ellipsoid technique (volume = length x width x depth x 0,523) and the 3D ultrasonography by VOCAL with 30° (6 delimitation planes) and 12° (15 delimitation planes).

The first ultrasonography assessment, denominated “day 0” corresponded to non-pregnant bitches, *i.e.* those animals evaluated before of the natural mating or that has mated but it wasn’t possible to identify the pregnancy. Other assessments were made every 7–14 days; which totalized 1 to 3 assessments per bitch. All animals were accompanied and grouped in three moments of time (day 0 = non-pregnant bitches; days 1^st^ to 20^th^ of pregnancy and days 21^st^ to 40^th^ of pregnancy). The following variables were evaluated with regard to renal volume of the bitches: 1) right side, ellipsoid technique, day 0; 2) right side, ellipsoid technique, 1^st^ - 20^th^ days; 3) right side, ellipsoid technique, 21^th^ - 40^th^ days; 4) right side, VOCAL 30, day 0; 5) right side, VOCAL 30, 1^st^ - 20^th^ days; 6) right side, VOCAL 30, 21^th^ - 40^th^ days; 7) right side, VOCAL 12, day 0; 8) right side, VOCAL 12, 1^st^ - 20^th^ days; 9) right side, VOCAL 12, 1^st^ - 20^th^ days; 10) left side, 2D, day 0; 11) left side, 2D, 1^st^ - 20^th^ days; 12) left side, 2D, 21^th^ - 40^th^ days; 13) left side, VOCAL 30, day 0; 14) left side, VOCAL 30, 1^st^ - 20^th^ days; 15) left side, VOCAL 30, 21^th^ - 40^th^ days; 16) left side, VOCAL 12, day 0; 17) left side, VOCAL 12, 1^st^ - 20^th^ days; 18) left side, VOCAL 12, 21^th^ - 40^th^ days.

To perform the ultrasound scans, the bitches were trichotomized ventrally from the sternum to the pubic portions and to the flanks and should be fasting for at least 6 hours. It was applied acoustic gel over the abdominal skin. The bitch was placed in right lateral decubitus for the left kidney evaluation and in left lateral decubitus for evaluation of contralateral kidney. Initially, for the renal volume acquisition, renal scan was performed in real time by 2D ultrasonography mode, until the complete longitudinal view of the kidney. The ideal longitudinal images were those that allowed the maximum measurement between the cranial and caudal poles with the renal pelvis visualization. A symmetrical form in “c” was formed by the renal sinus, pelvic diverticulum and renal ridge. Aiming the best contrast between the kidney and other abdominal organs, we used a predefined preset with frequency of 5 MHz, dynamic range of 75, harmonic mode and total gain of the device close to 100%. For the volumetric scanning, the 3D box was adjusted in order to embrace only the kidney in a fixed angle of 70° and fast scanning velocity in order to minimize the presence of transmissions artifacts resulting of the organs movimentation and of the animal respiratory frequency. We collected at least three volumes of each kidney by both examiners using the average of the best. The volumetric analysis was performed offline using the 1.03 version of the SonoView Pro software (Samsung Medison, Seoul, Korea).

**Figure 1:**
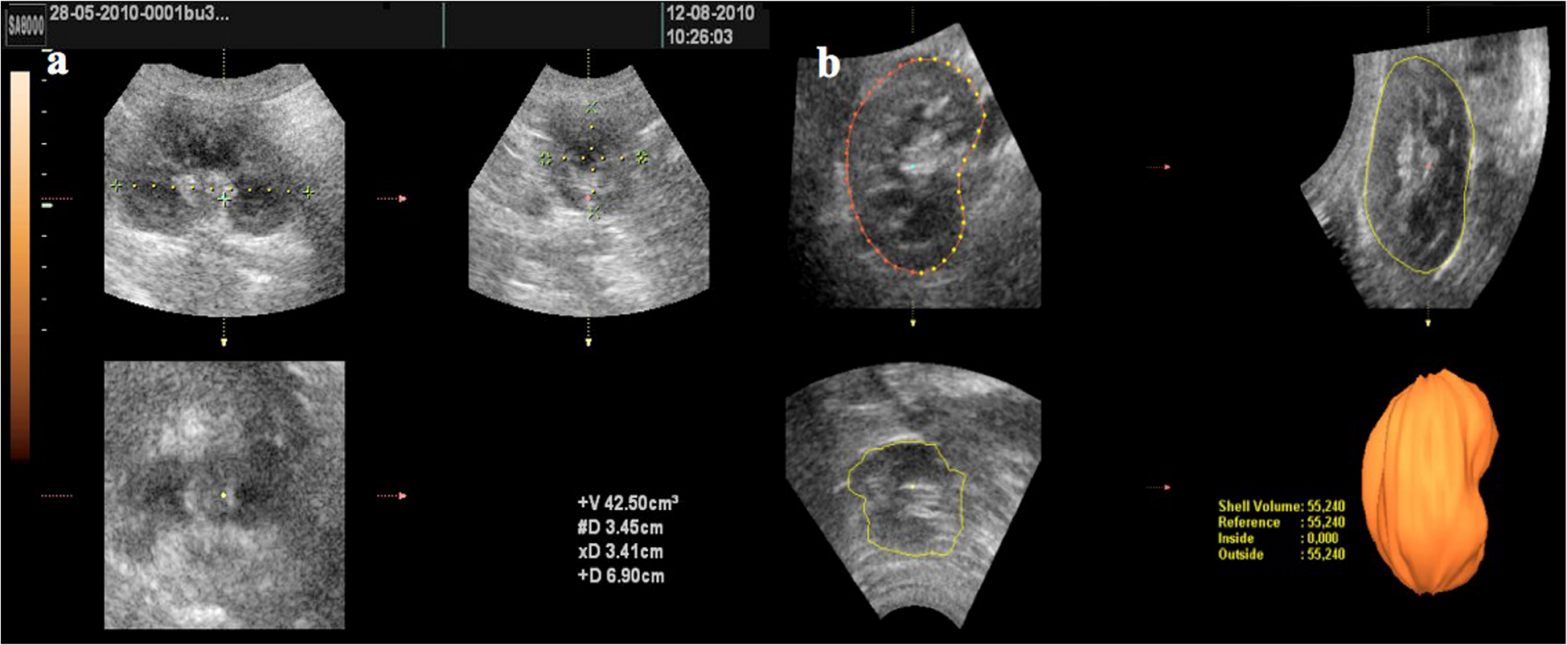
**Calculation of renal volume in pregnant bitches: a) calculation by two-dimensional method (ellipsoid): in plane A is measured length and in plan B the width and depth, which is automatically calculated the volume by the software, b) calculation by three-dimensional method (VOCAL): in plane A the kidney was rotated in “z” axis, and its external surface defined in manual mode, with rotation by 30° (6 plans) or 12 (15 plans).** At the end of the delimitation of the latter area the program automatically calculated the volume and provided a three-dimensionally reconstructed image. VOCAL: Virtual Organ Computer-aided AnaLysis

For each one of the 18 variables, the first examiner (DSM) performed two measurements with an interval of seven days, while the second examiner (RFFM) performed an third measure, “blinded” of the first examiner results. For each renal volume using the three techniques (2D, VOCAL 30° and VOCAL 12°) were performed three measurements which was considered the average of these for the statistical analysis. For the 2D ultrasonography calculation, it was used the multiplanar mode with automatic calculation by the ellipsoid formula in which the length was obtained in the longitudinal plane (A) and the width and depth in the axial plane (B) (Figure 1a). For the 3D ultrasonography calculation, realized fifteen days after the 2D ultrasonography assessment, we used the VOCAL method. Plan A was selected as reference being rotated around of axis “z” so that the kidney was in lengthwise position. After the selection of the key VOCAL, the calipers were placed in the upper and lower poles of the organ. It was firstly selected the rotation angle of 30° and manual delimitation mode. In this manner the renal area was manually delimited on its external surface in the six sequential planes, and the equipment provided the image reconstructed three-dimensionally as well as its volume in cm^3^ (Figure 1b). The same protocol was followed for the contralateral kidney. Was also analyzed the VOCAL method with an rotation angle of 12°, following the same steps previously described, 7 days after the previous phase.

All the data were collected in a specific protocol, later being transferred to an Excel 2007 spreadsheet (Microsoft Corp., Redmond, WA, USA). The statistical analysis was performed with the Statistical Package for the Social Science (SPSS) software version 17.0 for windows (SPSS Inc, Chicago, IL, USA). The statistical tests used were: ANOVA in the comparison of the renal volume (1^st^ measurement of the first examiner – DSM) regarding side, technique, moment of time besides the comparisons when necessary by the Turkey’s method; Shapiro-Wilk test to verify if the renal volume distribution (1^st^ measurement of the first examiner – DSM) regarding side, technique, moment of time is statistically normal; Pearson’s correlation coefficient (r) in order to evaluate the correlation between age (years) and the animals renal volumes at day 0, number of previous pregnancies and number of fetuses and difference of renal volume (21^st^ to 40^th^ days minus 1^st^ to 20^th^ day) of the animals; the intraclass correlation coefficient (ICC) to evaluate the inter- and intraobserver reproducibility. It was used the significance level (p) < 0.05 in all statistical analysis.

## Abbreviations

CT, Computed tomography; DSM, Débora Sartori Mendonça; MRI, Magnetic resonance imaging; RFFM, Rafael Fontoura Fernandes Moron; SD, Standard deviation; SPSS, Statistical package for the social science; VOCAL, Virtual organ computer-aided analysis.

## Competing interests

The authors declare that they have no competing interests.

## **Author’s contribution**

**DSM** – She realized the collect data. **RFFM** – He realized the interobserver reproducibility. **ALLM** – He realized the design of study **EAJ** – He realized the written and final analysis of the manuscript. **LMMN** – He realized the final analysis of the manuscript. **AFM** – He coordinated all steps of the study. All authors read and approved the final manuscript.

## Author’s information

**DSM** – Veterinary Physician, Masters in Science, Department of Obstetrics, Federal University of São Paulo (UNIFESP) **RFFM** – Veterinary Physician. **ALLM** – Veterinary Physician, PhD in Science, Department of Imaging Diagnostic, Federal University of São Paulo (UNIFESP). **EAJ** – Associate Professor, Department of Obstetrics, Federal University of São Paulo (UNIFESP). **LMMN** - Associate Professor, Department of Obstetrics, Federal University of São Paulo (UNIFESP). **AFM** – Full Professor, Department of Obstetrics, Federal University of São Paulo (UNIFESP).

## References

[B1] Luvoni GC Beccaglia M The prediction of parturition date in canine pregnancy Reprod Domest Anim 2006 41 27 32 10.1111/j.1439-0531.2006.00641.x 16420324

[B2] Riccabona M Nelson TR Pretorius DH Three-dimensional ultrasound: accuracy of distance and volume measurements Ultrasound Obstet Gynecol 1996 7 429 434 10.1046/j.1469-0705.1996.07060429.x 8807760

[B3] Nyland TG Mattoon JS Herrgesell EJ Wisner ER Nyland TG, Mattoon JS Urinary tract Small animal diagnostic ultrasound 2002 2 Philadelphia, PA: WB Saunders 93 127

[B4] Gilja OH Smievoll AI Thune N Matre K Hausken T Odegaard S Berstad A In vivo comparison of 3D ultrasonography and magnetic resonance imaging in volume estimation of human kidneys Ultrasound Med Biol 1995 21 25 32 10.1016/0301-5629(94)00082-4 7754576

[B5] Riccabona M Fritz GA Schollnast H Schwarz T Deutschmann MJ Mache CJ Hydronephrotic kidney: pediatric three-dimensional US for relative renal size assessment–initial experience Radiology 2005 236 276 283 10.1148/radiol.2361040158 15955855

[B6] Barr FJ Holt PE Gibbs C Ultrasonographic measurement of normal renal parameters J Small Animal Practice 1990 31 180 184 10.1111/j.1748-5827.1990.tb00764.x

[B7] Felkai CS Vörös K Vrabély T Karsai F Ultrasonographic determination of renal volume in the dog Vet Radiol Ultrasound 1992 33 292 296 10.1111/j.1740-8261.1992.tb00146.x

[B8] Tedesco GD Bussamra LC Araujo E Britto IS Nardozza LM Moron AF Aoki T Reference range of fetal renal volume by three-dimensional ultrasonography using the VOCAL method Fetal Diagn Ther 2009 25 385 391 10.1159/000236151 19786784

[B9] Nyland TG Kantrowitz BM Fisher P Olander HJ Hornof WJ Ultrasonic determination of kidney volume in the dog Vet Radiol 1989 30 174 180 10.1111/j.1740-8261.1989.tb00771.x

[B10] Pang BS Kort BC Ying M Three-dimensional ultrasound volumetric measurements: is the largest number of image planes necessary for outlining the region-of-interest? Ultrasound Med Biol 2006 32 1193 1202 10.1016/j.ultrasmedbio.2006.04.012 16875954

[B11] Raine-Fenning NJ Clewes JS Kendall NR Bunkheila AK Campbell BK Johnson IR The interobserver reliability and validity of volume calculation from three-dimensional ultrasound datasets in thein vitrosetting Ultrasound Obstet Gynecol 2003 21 283 291 10.1002/uog.61 12666225

[B12] Martins WP Ferriani RA Ferreira AC The reproducibility of endometrial volume measurements using the VOCAL - the importance of the step rotation Rev Bras Ginecol Obstet 2006 28 38 43 10.1590/S0100-72032006000100007

[B13] Konde LJ Wrigley RH Park RD Lebel JL Ultrasonographic anatomy of the normal canine kidney Vet Radiol 1984 25 173 178 10.1111/j.1740-8261.1984.tb02138.x

[B14] Sampaio KM Araujo RB Ultrasound features both linear and estimated volume of kidneys in dogs Arq Bras Med Veter Zootec 2002 54 248 254 10.1590/S0102-09352002000300005

[B15] Christensen T Klebe JG Bertelsen V Hansen HE Changes in renal volume during normal pregnancy Acta Obstet Gynecol Scand 1989 68 541 543 2520811

